# Preparation of High-Temperature Resistant Aerogels by Incorporating Aluminum Sol into Composite Silica Sources Using Ambient Pressure Drying

**DOI:** 10.3390/polym16162296

**Published:** 2024-08-14

**Authors:** Shuai Gao, Zeqi Cao, Kai Liu, Shuning Liu, Wanjun Pang, Hongyi Jiang

**Affiliations:** School of Materials Science and Engineering, Wuhan University of Technology, Wuhan 430070, China; gaos@whut.edu.cn (S.G.); caozeqi@whut.edu.cn (Z.C.); 317482@whut.edu.cn (K.L.); shuning@whut.edu.cn (S.L.); pwj2000@whut.edu.cn (W.P.)

**Keywords:** silica aerogel, aluminum sol, high-temperature resistance, ambient pressure drying

## Abstract

To reduce production costs and enhance the high-temperature resistance of SiO_2_ aerogels, an aluminum-doped silica aerogel (ASA) was successfully prepared using the sol-gel method and atmospheric drying method. The composite silica sources included TEOS and inexpensive acidic silica sol, while the aluminum source was aluminum sol. The study investigated the influence of the molar ratio of acidic silica sol to TEOS, Al/Si, and calcination temperature on the composition, structure, and high-temperature resistance of the ASA. The results indicate that a sample with an acidic silica sol to TEOS molar ratio of 0.8 achieved a specific surface area of 683.204 m^2^·g^−1^. The Al/Si molar ratio significantly impacted the high-temperature resistance of the ASA, with the sample having a molar ratio of 0.02 Al/Si displaying the highest specific surface area of 705.956 m^2^·g^−1^ at 600 °C. Moreover, this surface area remained at 273.099 m^2^·g^−1^ after calcination at 1000 °C, notably higher than the sample without aluminum sol (16.082 m^2^·g^−1^). Mechanism analysis indicated that the addition of aluminum sol to the SiO_2_ aerogel inhibited phase transitions, and both acidic silica sol and aluminum sol particles enhanced the aerogel structure, contributing to a marked improvement in high-temperature resistance.

## 1. Introduction

Silica aerogels are nano-porous materials characterized by their intricate three-dimensional framework and exhibit remarkable properties stemming from their high specific surface area, pronounced porosity, and low density [1,2]. Notably, these features confer upon silica aerogels an exceptionally low thermal conductivity, typically falling within the range of 0.005 to 0.1 W/m·k [3]. Since their pioneering synthesis by Kistler in 1931, silica aerogels have undergone extensive examination [4]. In recent years, the preparation of silica aerogels has evolved into a well-established technology with applications spanning aerospace, building energy efficiency, and the chemical industry [5,6,7].

The sol-gel method is the most commonly used method for the preparation of silica aerogels, and the precursors include organic silanes (e.g., tetraethyl orthosilicate, methyl trimethoxysilane, vinyl trimethoxysilane, and vinyl methyl dimethoxysilane) [8,9,10] and inorganic silica sources (e.g., water glass and silica sol) [11,12]. However, organic silanes are expensive and volatile. Some silanes and their hydrolysis products are toxic, posing safety hazards and limiting their industrial production. The preparation of silica aerogel from sodium silicate is typically achieved through cation exchange to remove Na^+^ from the water glass or using distillation to remove Na^+^ from the gel. However, these methods are time-consuming and involve complex recovery of cation exchange resins. Currently, tetraethyl orthosilicate (TEOS) is the most mature preparation process with TEOS as the precursor. Still, aerogels prepared from a single TEOS are usually susceptible to cracks and breakage after atmospheric pressure drying (APD) [13]. The integrity of aerogels can be effectively maintained using freeze drying (FD) or supercritical drying (SCD), but both have cost and safety limitations [14].

Furthermore, the unique nanostructure of silica aerogels contributes to their high surface activity, resulting in an enlargement of nanoparticle size and the disruption of the pore structure in high-temperature environments. Specifically, sintering becomes problematic at temperatures exceeding 650 ℃, thereby limiting the broader applicability of silica aerogels [15]. To enhance the stability of silica aerogels in high-temperature conditions, the introduction of high-temperature resistant fibers (e.g., mullite whiskers, alumina powder, and glass fibers) represents a potential solution [16,17,18]. However, achieving uniform fiber dispersion proves challenging when employing mechanical or ultrasonic dispersion methods. Moreover, the incorporation of fibers generally leads to increased density, thermal conductivity, and internal stress within the aerogel, which can have adverse effects on its overall performance. A more effective approach for improving the high-temperature resistance of silica aerogels involves enhancing the bonding, energy, and stability of the ionic crystal structure [19]. The inclusion of high melting point metal oxide elements (e.g., alumina, zirconia, titanium dioxide, vanadium dioxide, and zinc dioxide) into silica aerogel precursors can significantly boost their thermal stability, with SiO_2_-Al_2_O_3_ composite aerogels displaying exceptional resistance to high temperatures [15]. Organo aluminum alkoxides (e.g., ASB and AIP) are commonly employed as aluminum sources for aerogel preparation, typically utilizing the SCD method along with chelating agents to control the chemical reaction rate [20,21]. In contrast, the use of water and inorganic aluminum salts (e.g., Al(NO_3_)_3_·9H_2_O and AlCl_3_·6H_2_O) with the APD method offers a safer and more straightforward alternative [11,22]. However, it is worth noting that inorganic aluminum salts are susceptible to precipitation in alkaline environments during the gelation process, resulting in the loss of aluminum elements and an uneven distribution. Consequently, a promising avenue of research revolves around reducing the cost of raw materials and utilizing a stable aluminum source to produce composite aerogels with excellent high-temperature performance with the APD method.

The fundamental structural unit of acidic silica sol particles is the silica-oxygen tetrahedron (SiO_4_). These tetrahedra are interconnected by siloxane bonds (Si-O-Si), forming spherical structures with double layers of varying degrees of polymerization. The silanol groups on the particle surfaces are primarily protonated, resulting in positively charged Si-OH^2+^ groups. Our research team successfully prepared monolithic, crack-free silica aerogels with a density of 0.130 g/cm^3^ by employing TEOS and basic silica sol as co-precursors using the APD method [23]. However, due to the high alkalinity (pH = 9.9) of the basic silica sol, its application is constrained, requiring dilution for optimal use. The preparation of SiO_2_ aerogels using acidic silica sol as a silicon source has been reported but suffers from the disadvantages of difficult molding and low comparative area [24]. Aluminum sol, also known as alumina sol or boehmite sol, finds widespread utility in industries such as petrochemicals, textiles, and paper. Aluminum sol exhibits typical colloidal characteristics, including a bilayer structure comprising adsorption and diffusion layers surrounding a colloidal nucleus. In addition, the surface of the pellet is rich in active -OH groups. The positively charged adsorbent layer prevents particle aggregation through electrostatic repulsion, thereby maintaining the stability of the sol [25]. At present, the synthesis of silica aerogels using TEOS and acidic silica sol as co-precursors remains an unexplored territory, with limited research dedicated to the preparation of SiO_2_-Al_2_O_3_ composite aerogels utilizing aluminum sol as an inorganic aluminum source.

This study aims to reduce the production cost of aerogels by incorporating inexpensive inorganic silicon sources to replace part of the organic silicon sources. Additionally, we use a stable aluminum source to obtain composite aerogel materials with excellent high-temperature resistance using a simple preparation method. In our current investigation, we have successfully synthesized aluminum-doped SiO_2_-Al_2_O_3_ composite aerogels (ASAs) utilizing tetraethyl orthosilicate (TEOS) in combination with acidic silica sol as the composite silicon source and aluminum sol as the inorganic aluminum source. These composite aerogels were prepared through a combination of sol-gel and atmospheric pressure drying (APD) methods. Our study sought to understand the impact of various factors, including the molar ratio of acidic silica sol to TEOS in the precursor, the Al/Si molar ratio, and the calcination temperature, on the structural and property characteristics of these composite aerogels. Furthermore, we explored the heat-resistant mechanism exhibited by ASAs. By substituting a portion of TEOS with acidic silica sol, we have effectively managed to reduce production costs. Moreover, the use of inexpensive inorganic aluminum sources and the utilization of a straightforward and safe atmospheric pressure drying process in the preparation of SiO_2_-Al_2_O_3_ composite aerogels present a novel approach that holds promise for promoting industrial-scale production of these materials.

## 2. Materials and Methods

### 2.1. Materials and Preparation

The aluminum-doped silica aerogel (ASA) was synthesized via a combination of sol-gel and atmospheric pressure drying processes. TEOS (≥99.0%, Sinopharm Chemical Reagent Corporation, Beijing, China) was blended with ethanol (≥99.0%, Sinopharm Chemical Reagent Corporation, China) and deionized water. To adjust the pH to a range of 2–3, 0.5 M HCl was added. The mixing ratio was as follows: TEOS: ethanol: water = 1:9:2. The mixture was stirred at room temperature for 3 h to ensure complete hydrolysis of TEOS. Subsequently, various proportions of acidic silica sol (solid content: 30.8%, pH = 4.0, Guangzhou Fur Chemical Technology Co., Ltd. Guangzhou, China) were added to the hydrolyzed solution under continuous stirring. The molar ratios of acidic silica sol to TEOS varied, including 0, 0.2, 0.4, 0.6, 0.8, 1.0, and 1.2, and these were denoted as SA-0 to SA-1.2. The molar ratio is defined as the ratio of SiO_2_ in the acidic silica sol to Si in TEOS. After 10 min of stirring, different ratios of aluminum sol (solid content: 20%, pH = 4.3, Hangzhou Jiupeng New Materials Co., Ltd., Hangzhou, China) were introduced into the mixture. The molar ratios of aluminum sol to silicon (referred to as Al/Si) were 0.02, 0.04, 0.06, 0.08, 0.10 and 0.12, and these were labeled as ASA-0.02 to ASA-0.12. Following thorough mixing for 10 min, 1 M ammonia (26–28%, Sinopharm Chemical Reagent Company, China) was slowly added drop by drop to adjust the pH to 7. After an additional 5 min of stirring, the mixed sol was transferred to a polyethylene mold and allowed to gel at room temperature. Once gelation was complete, the gel was aged at 45 °C for 12 h and subsequently immersed in a solution of ethanol and TEOS (in a 4:1 ratio) for 24 h to enhance its structural integrity. The gels then underwent two solvent exchanges using n-hexane (≥97%, Aladdin Industries, Shenzhen, China), with each exchange taking place every 12 h. Afterward, the gel was subjected to hydrophobic modification by immersing it in a mixture of TMCS (≥99.5%, Sinopharm Chemical Reagent Corporation, China) and n-hexane at a volume ratio of 1:9 for 24 h. Given the presence of unreacted TMCS and HCl generated during the reaction in the pore space of the gel, the gel underwent four rounds of cleaning with n-hexane, with the solvent being replaced every 12 h. Finally, after hydrophobic modification, the gel underwent two solvent exchanges, with a change in solvent every 12 h. Subsequently, the hydrophobically modified gel was dried at 60 °C for 12 h, resulting in the formation of SiO_2_-Al_2_O_3_ composite aerogel.

### 2.2. Characterization

All the components were homogeneously mixed and then allowed to stand. The beaker was tilted at 45°, and the gel was completed when the gel did not flow in the beaker. The time taken was denoted as the gelation time of the aerogel. The bulk density of the aerogel was calculated from the mass-to-volume ratio measured using the drainage method. The specific surface area and pore size distribution of the aerogels were analyzed using a fully automated specific surface area and pore size analyzer (JW-BK112, JWGB, Beijing, China) using the Brunauer–Emmett–Teller (BET) and Barrett–Joyner–Halenda (BJH) methods, and the samples were degassed at 150 °C for 3 h before the testing. We used a scanning electron microscope/energy dispersive X-ray spectrometer (SEM/EDS, JEM-2100 F, JEOL, Akishima, Japan) to observe the micromorphology and elemental distribution of the samples. An X-ray diffractometer (XRD, D/MAX-RB, Rigaku Corporation, Tokyo, Japan) was used to characterize the composition of the physical phases in the samples. A Fourier transform infrared spectrometer (FTIR, Nexus, Nicolet, Waltham, MA, USA) was used to analyze the chemical composition of the aerogels. Raman spectra (LabRAM Odyssey, Horiba Scientific, Paris, France) were used to analyze SiO_4_ tetrahedral networks of gels. A simultaneous thermal analyzer (TG-DTA, STA449F3, NETZSCH, Bamberg, Germany) was used to analyze the thermal stability of the samples under the test conditions of an air atmosphere with a temperature increase rate of 10 °C/min.

## 3. Results and Discussion

### 3.1. Physical Properties of the SA

Table 1 provides an overview of how the gel time, bulk density, and pore structure of each sample vary with different molar ratios of acidic silica sol to TEOS. It is evident from the table that the gelation time exhibits an initial decrease and subsequently an increase with an increase in the addition of acidic silica sol. Notably, the sample SA-0.6 demonstrates the shortest gelation time recorded at 51 min. Simultaneously, the bulk density shows a progressive increase, ranging from 0.122 g·cm^−3^ to 0.178 g·cm^−3^. This observed phenomenon can be attributed to the presence of acidic silica sol, which plays a crucial role in promoting the gelation process. The substantial concentration of reactive -OH groups on the particle surfaces provides numerous topographical nucleation sites for gelation, resulting in a shorter gelation time. However, as the molar ratio of acidic silica sol to TEOS continues to increase, the increased water content in the system creates a greater distance between hydrolyzed reactive Si-OH groups. This extended distance delays the formation of the gel network. Furthermore, a higher molar ratio of acidic silica sol to TEOS leads to a more concentrated concentration of system reactants, resulting in a higher concentration of SiO_2_ within the network skeleton and an overall increase in aerogel density. Additionally, the excess water present in the gel pores as a consequence of the increased molar ratio of acidic silica sol to TEOS can cause the collapse of the aerogel skeleton during the drying process, further contributing to the increased aerogel density.

The quantity of acidic aluminum sol added significantly influenced the pore structure of the aerogels. As the molar ratio of acidic aluminum sol to TEOS increased, the specific surface area of SA initially increased and then decreased. SA-0.6, in particular, exhibited the highest specific surface area at 754.243 m^2^·g^−1^, along with a pore volume of 2.806 cm^3^·g^−1^ and an average pore diameter of 14.880 nm. This change in specific surface area mirrors the behavior of the aerogel density, with both phenomena attributed to similar causes. First, the addition of acidic silica sol particles provided an abundance of nucleation sites during the gelation process, enhancing the cross-linking degree of the aerogel skeleton. This increase in cross-linking resulted in an expanded specific surface area and a more refined pore size. Conversely, an excessive quantity of acidic aluminum sol introduced excess moisture into the gel pores. This surplus moisture amplified the capillary effects within the gel skeleton, which, during the drying process, could lead to the collapse of the gel pore structure. This collapse, in turn, caused a decrease in the specific surface area and an increase in pore size.

**Figure 1:**
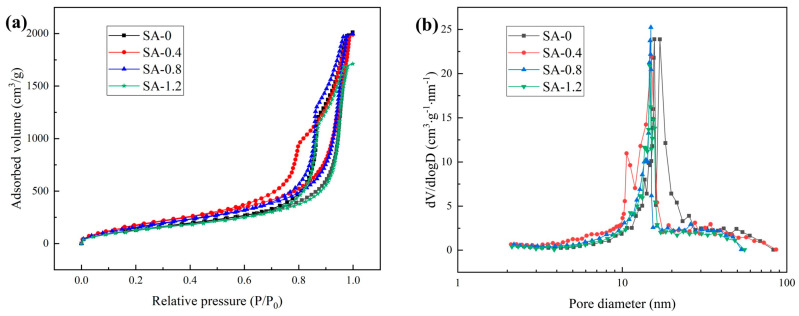
Pore structure analysis of SA-0, SA-0.4, SA-0.8, and SA-1.2: (**a**) N_2_ adsorption–desorption isotherms; (**b**) Pore diameter distributions.

The N_2_ adsorption–desorption results of the SA samples, following drying at atmospheric pressure, are presented in Figure 1. In Figure 1a, the adsorption–desorption isotherms demonstrate that samples with varying molar ratios of acidic silica sol to TEOS exhibit type IV isothermal adsorption curves, indicating the typical mesoporous nature of the SA samples [26]. The initial increase in adsorption occurs gradually when the relative pressure (P/P_0_) is less than 0.6, primarily representing the monomolecular layer adsorption of N_2_. As the relative pressure increases, there is a rapid growth in adsorption, signifying the occurrence of multimolecular layer adsorption and capillary condensation. Notably, H3-type hysteresis lines are evident in all prepared SA samples, indicating the presence of numerous slit-type mesopores within the samples [27]. In Figure 1b, the pore size distribution curve of the SA samples reveals that the majority of pores fall within the range of 5–30 nm. The introduction of an acidic aluminum sol, compared to SA-0, leads to a refinement of pore size. However, as the content of acidic silica sol increases, the pore size expands, accompanied by an increase in the number of pores. For example, in comparison to SA-0.4, SA-0.8 exhibits a decrease in specific surface area from 730.257 m^2^·g^−1^ to 683.204 m^2^·g^−1^. Conversely, the pore volume increases from 3.077 m^2^·g^−1^ to 3.090 m^2^·g^−1^, and the average pore size increases from 16.856 nm to 18.094 nm. Given that the addition of acidic silica sol in the SA-0.8 sample results in a reduction in production costs and only minor changes in pore structure compared to SA-0.4 and SA-0.6, our subsequent discussion will primarily concentrate on the SA-0.8 sample.

### 3.2. Morphology, Crystalline and Chemical Structure of the SAs

**Figure 2:**
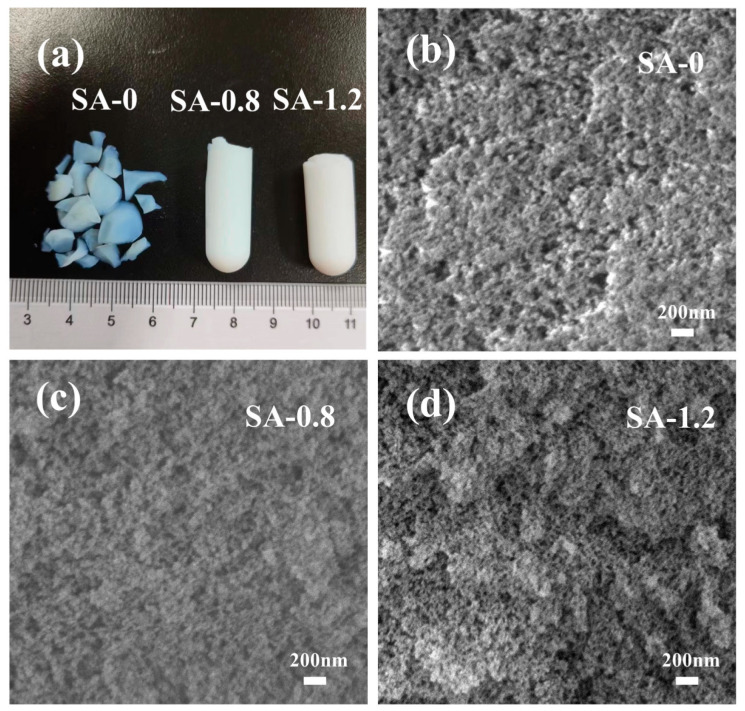
Morphology (**a**) and SEM images (**b**–**d**) of SA-0, SA-0.8, and SA-1.2.

The samples dried at atmospheric pressure with acidic silica sol to TEOS molar ratios of 0, 0.8, and 0.12 are shown in Figure 2a. The SA-0 samples were in the form of crumbled blocks, and the SA-0.8 and SA-1.2 aerogels were molded intact and free of cracks. This finding suggests that the addition of acidic silica sol can improve the strength of the SiO_2_ aerogel skeleton, which is beneficial to maintain the integrity of the blocks after drying. Figure 2b–d shows the SEM images of the three samples, separately. From the figures, it can be seen that the SiO_2_ aerogel consists of a porous material with a large number of nanopores and nanoparticles, in which the crosslinked nano-spherical particles polymerize to form a continuous 3D network skeleton. With the addition of acidic sol, the particles of SA-0.8 samples are more tightly packed, and the pore size of the aerogel is refined and the pore size distribution is more uniform. When the molar ratio of acidic silica sol to TEOS was 1.2, the particles piled up to form clusters. In addition, the agglomeration phenomenon occurred, and the pore size and uniformity deteriorated. Acidic silica sol provides nucleation sites during the gelation process and plays the role of enhancing the skeleton to optimize the pore structure. While the excess acidic silica sol particles themselves undergo dehydration and condensation reactions, these larger silica sol particles are more likely to form stacked clusters.

**Figure 3:**
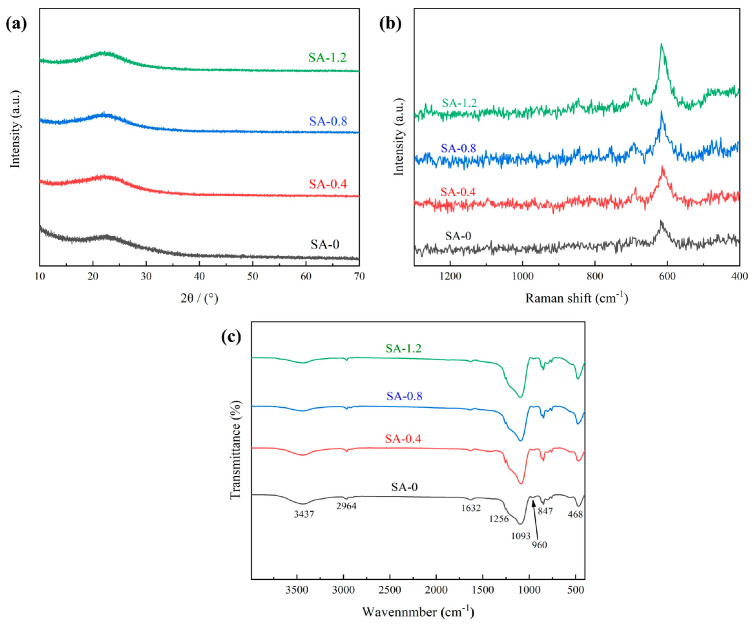
XRD pattern (**a**), Raman spectra (**b**) and FTIR detection (**c**) of samples with different acidic silica sol to TEOS molar ratios.

In Figure 3a, XRD spectra of acidic silica sol with TEOS at various molar ratios are presented. These spectra reveal a diffuse peak within the 2θ range of 20–25° for all four sample groups, corresponding to the characteristic peak of SiO_2_. This observation suggests that the SA samples maintain an amorphous nature, and the addition of acidic silica sol does not alter the crystal structure of the aerogel. Figure 3b shows the Raman spectra of each sample. In the 400–700 cm^−1^ range, these spectra correspond to the bending vibration of Si-O-Si, and the peaks within this range increase with the higher acidic silica sol content. This increase signifies an enhancement in the polymerization of the SiO_2_ skeleton within the aerogel. Notably, the vibrational peaks associated with Q^n^ (the fundamental structural unit of SiO_4_ tetrahedra) do not appear more obviously in the 700–1300 cm^−1^ band. This lack of variation indicates that the composite silicon source underwent sufficient reaction during the gelation and aging process, resulting in the formation of a complex three-dimensional network skeleton. Figure 3c displays the FTIR spectrum of the sample following hydrophobic modification and atmospheric pressure drying. Notably, the absorption peaks of SA-0 and the sample after the addition of acidic silica sol exhibit a high degree of similarity. In particular, the more prominent absorption peaks at 1093 cm^−1^ and 468 cm^−1^ correspond to the antisymmetric stretching and bending vibrations of Si-O-Si groups, indicating that the SiO_2_ aerogel skeleton primarily consists of Si-O-Si bonds [28]. The absorption peaks at 2964 cm^−1^, 1256 cm^−1^, and 847 cm^−1^, corresponding to -CH_3_ and Si-C [29], are a result of the substitution of hydrophilic -OH groups in the wet gel skeleton by hydrophobic -CH_3_ groups during the modification process using TMCS. Additionally, the absorption peaks at 3437 cm^−1^ and 1632 cm^−1^ are attributed to the stretching vibration of -OH and the bending vibration of H-O-H in H_2_O [30], while the peak at 960 cm^−1^ corresponds to the antisymmetric stretching vibration of Si-OH [31]. These observations indicate the presence of residual adsorbed water within the aerogel skeleton and a small quantity of -OH groups even after hydrophobic modification.

### 3.3. Physical Properties of the ASA

In Table 2, the impact of different aluminum sol contents on composite aerogel time, density, and pore structure is presented. An increase in aluminum sol content corresponds to an extended gelation time, rising from 55 min to 125 min. Simultaneously, the density of the sample increases from 0.138 g·cm^−3^ to 0.223 g·cm^−3^, aligning with findings from a prior study. Additionally, the specific surface area of the aerogel exhibits a declining trend as the aluminum sol content increases. Two primary reasons account for the reduction in specific surface area. Firstly, both acidic silica sol particles and aluminum sol particles carry a negative charge on their surfaces, creating a repulsive force in the sol system after mixing due to their similar charges. This impedes the gel reaction, contributing to the observed increase in gel time. Secondly, the addition of aluminum sol partially dilutes the composite sol, leading to increased spacing between active Si-OH after TEOS hydrolysis, ultimately resulting in volume contraction during atmospheric pressure drying. This process contributes to the densification of aerogel samples, explaining the decrease in aerogel pore volume and average pore size with increasing aluminum sol content.

**Figure 4:**
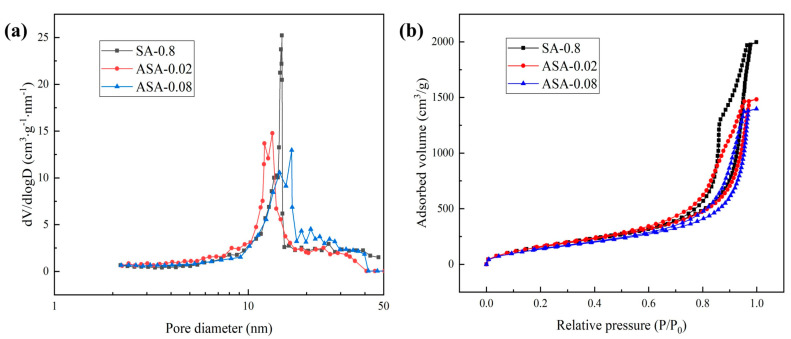
Pore structure analysis of SA-0.8, ASA-0.02, and ASA-0.08: (**a**) Pore diameter distributions; (**b**) N_2_ adsorption–desorption isotherms.

Figure 4a,b illustrates the pore size distribution and adsorption–desorption isotherms of composite aerogels with varying aluminum sol contents. The figure reveals that the pore size distribution is narrow, and concentrated within the 10–30 nm range. Additionally, the number of pores decreases after the addition of aluminum sol. Specifically, when the molar ratio of Al/Si is 0.08, the pore size of ASA-0.08 samples increases within the range of 15–30 nm. This change arises from the electrostatic repulsion between identically charged particles when the acidic silica sol is mixed with the aluminum sol. This repulsion hinders the gelation reaction, resulting in an aerogel with reduced pore volume and specific surface area, as well as a decrease in average pore diameter. Additionally, the introduction of aluminum sol particles alters the interactions among the original acidic silica sol particles. The aluminum sol particles interact with silicate ions in the silica sol, which modifies the dispersion of the sol particles.

In the isothermal adsorption–desorption curve of Figure 4b, we further elucidate the changes in the pore structure of the composite aerogels. An increase in the Al/Si molar ratio results in a decrease in the N_2_ adsorption ability of the corresponding aerogel samples. This decrease is particularly pronounced in the high relative pressure range. Notably, ASA-0.02 and ASA-0.08 samples exhibit weaker adsorption ability, indicating a reduction in the number of pores after drying at atmospheric pressure. Observing the hysteresis ring, it is apparent that the addition of aluminum sol results in a small hysteresis ring, signifying a lower number of internal pores and weaker capillary coagulation. This outcome is attributed to an excess of aluminum sol, leading to increased sample shrinkage during drying and subsequently decreasing the adsorption capacity of the sample for N_2_ as the pore size decreases.

### 3.4. Morphology, Crystalline, and Chemical Structure of the ASAs

**Figure 5:**
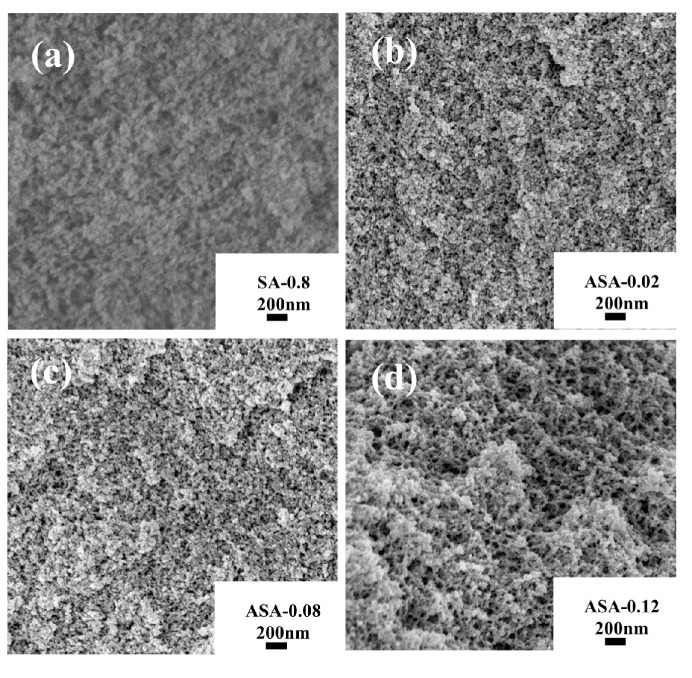
SEM images of samples with different Al/Si molar ratios: SA-0.8 (**a**); ASA-0.02 (**b**); ASA-0.8 (**c**); ASA-0.12 (**d**).

Figure 5 shows the SEM micrographs of samples with different Al/Si molar ratios. The images reveal that adding aluminum sol leads to all samples displaying a three-dimensional network structure. The ASA-0.02 sample has well-developed pores with uniformly distributed skeletal particles. However, with increasing aluminum sol content, the ASA-0.08 and ASA-0.12 samples exhibit particle agglomeration within the framework, forming small, unevenly distributed clusters.

**Figure 6:**
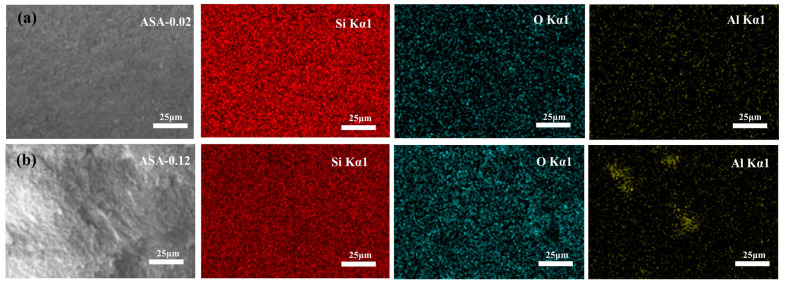
EDS surface analysis diagram of samples: ASA-0.02 (**a**); ASA-0.12 (**b**).

The elemental distribution of Si, O, and Al in the ASA-0.02 and ASA-0.12 samples was characterized using EDS, as shown in Figure 6. The EDS spectra demonstrate a uniform distribution of elements within the aerogel framework for the ASA-0.02 sample. In the ASA-0.12 sample, Si and O elements are evenly distributed, while the Al element shows slight aggregation in certain areas. The electrostatic repulsion between the acidic silica sol and aluminum sol particles increases with higher aluminum sol content, which hinders the gelation reaction and leads to the non-uniform distribution of Al. The test results indicate that the Al/Si molar ratios for the ASA-0.02 and ASA-0.12 samples are 0.006 and 0.027, respectively, both of which are lower than the theoretical values. This suggests that not all aluminum from the aluminum sol was successfully incorporated into the aerogel framework, with some aluminum being lost during the solvent exchange process.

**Figure 7:**
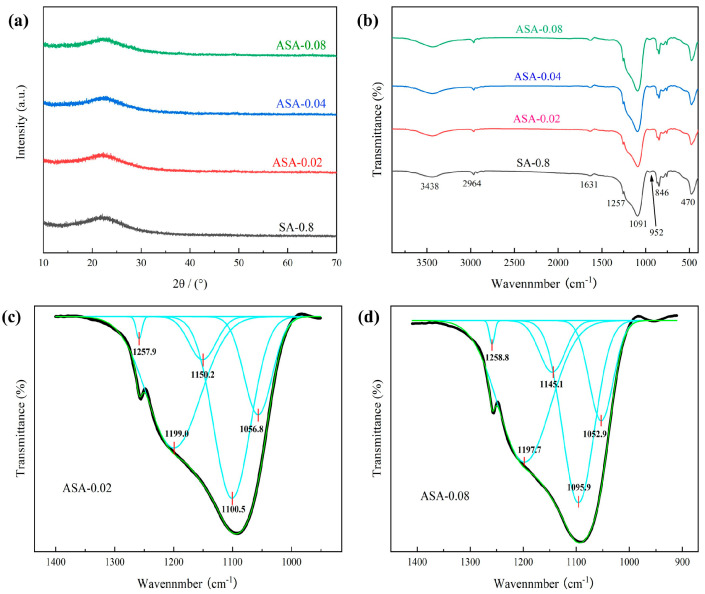
XRD pattern (**a**,**b**) FTIR detection of samples with different Al to Si molar ratios, curve–fitting for (**c**) ASA-0.02 and (**d**) ASA-0.08.

Figure 7 presents the XRD and FTIR spectra of samples with varying amounts of aluminum sol added. As shown in Figure 7a, all samples are amorphous. Instead, a diffuse peak between 2θ = 20–25° is evident, corresponding to the characteristic peak of crystalline SiO_2_. The XRD indicates that the sample is X-ray amorphous for this way of measurement. The Al/Si molar ratio’s influence on ASA composition was further examined using FTIR analysis at 400 and 4000 cm^−1^ as depicted in Figure 7b. The characteristic peaks of the curves show no significant changes with increasing aluminum sol content. For a more in-depth investigation into the chemical structure, we performed curve fitting for ASA-0.02 and ASA-0.08 in the range of 900 to 1400 cm^−1^ as illustrated in Figure 7c,d. Distinct absorption peaks are observed at 1258 and 1199 cm^−1^, corresponding to -CH_3_ absorption, while 1100 cm^−1^ signifies the tensile vibration of Si-O. Additionally, 1150 cm^−1^ represents the Al-O and AlO-H bonding vibrations of boehmite, and 1056 cm^−1^ is the absorption peak of Si-O-Al bonding [32]. Despite similar Si-O-Al absorption peak intensities in ASA-0.02 and ASA-0.08, the latter exhibits a higher intensity in the boehmite absorption peak. This suggests that some aluminum from the aluminum sol has been incorporated into the SiO_2_ skeleton, forming Si-O-Al bonds, while the remaining aluminum persists in the form of boehmite. Notably, the XRD pattern does not clearly show boehmite characteristic peaks due to its low content in the added aluminum sol. Additionally, an increase in aluminum sol content does not significantly elevate the number of Si-O-Al bonds, indicating that excess aluminum sol coexists in the aerogel skeleton in the form of boehmite.

### 3.5. Thermal Stability of the ASAs

The specific surface area of the composite aerogel samples after various high-temperature treatments is presented in Table 3, with the temperature of the uncalcined sample recorded as 25 °C. The specific surface area of the SA-0.8 sample decreases significantly with the increase in calcination temperature, reaching only 16.083 m^2^·g^−1^ at 1000 °C. The addition of aluminum sol notably enhances the sample’s stability at high temperatures. For the ASA-0.02 sample, with an Al/Si molar ratio of 0.02–0.08, the specific surface area initially increases and then decreases with the rise in calcination temperature. The maximum specific surface area of the ASA-0.02 sample is 705.956 m^2^·g^−1^ at 600 °C. After treatment at 1000 °C, a relatively high specific surface area of 273.099 m^2^·g^−1^ is retained. The specific surface area of ASA-0.1 and ASA-0.12 samples decreases with the increase in temperature, but they exhibit relative stability at 600 and 800 °C. Overall, these results suggest that the introduction of aluminum sol enhances the thermal stability of the composite aerogel samples.

**Figure 8:**
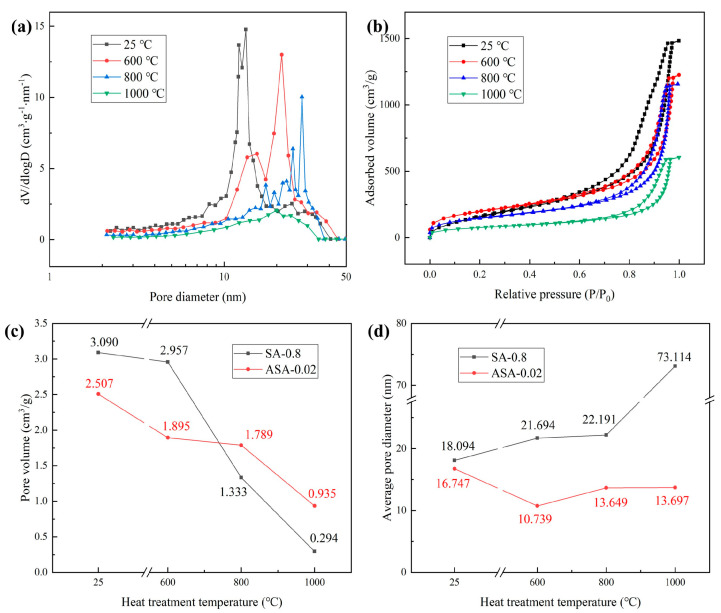
N_2_ adsorption–desorption results of ASA-0.02 calcined at different temperatures: (**a**) Pore diameter distribution; (**b**) N_2_ adsorption–desorption isotherms; Comparison with SA-0.8 and ASA-0.02: (**c**) Pore volume; (**d**) Average pore diameter.

Figure 8a–d depict the pore size distribution, N_2_ adsorption–desorption isothermal curves, pore volume, and average pore size of ASA-0.02 samples after calcination at various temperatures. In Figure 8a, it is evident that the sample’s pore size increases and decreases with the rise in calcination temperature. Simultaneously, the pore size distribution widens, and the pore size corresponding to the concentrated area increases. This is attributed to the deformation of the aerogel skeleton under thermal stress, leading to the interconnection of smaller pores and an overall increase in pore size distribution. However, at 600 and 800 °C, the samples still maintain a higher number of pores, with mesopores distributed in the range of 10–50 nm. The N_2_ adsorption–desorption isotherms in Figure 8b reveals a weakened N_2_ adsorption capacity after high-temperature calcination, especially evident after treatment at 1000 °C. This suggests a gradual reduction in nanopores after high-temperature treatment. Figure 8c illustrates the curves of pore volume variation with calcination temperature for the SA-0.8 and ASA-0.02 samples. The results indicate a decrease in pore volume with increasing calcination temperature for both. SA-0.8 experiences a sharp decrease above 600 °C, reaching only 0.294 cm^3^/g at 1000 °C. In contrast, ASA-0.02 maintains a more stable pore volume between 600 and 800 °C, registering 0.935 cm^3^/g at 1000 °C, surpassing SA-0.8. In Figure 8d, the average pore size of the SA-0.8 sample increases with temperature, reaching 73.114 nm at 1000 °C, signifying damage to the SiO_2_ aerogel network and a subsequent increase in pore size. Conversely, the ASA-0.02 sample’s average pore size remains in the range of 13–17 nm, preserving the mesoporous structure after high-temperature calcination.

**Figure 9:**
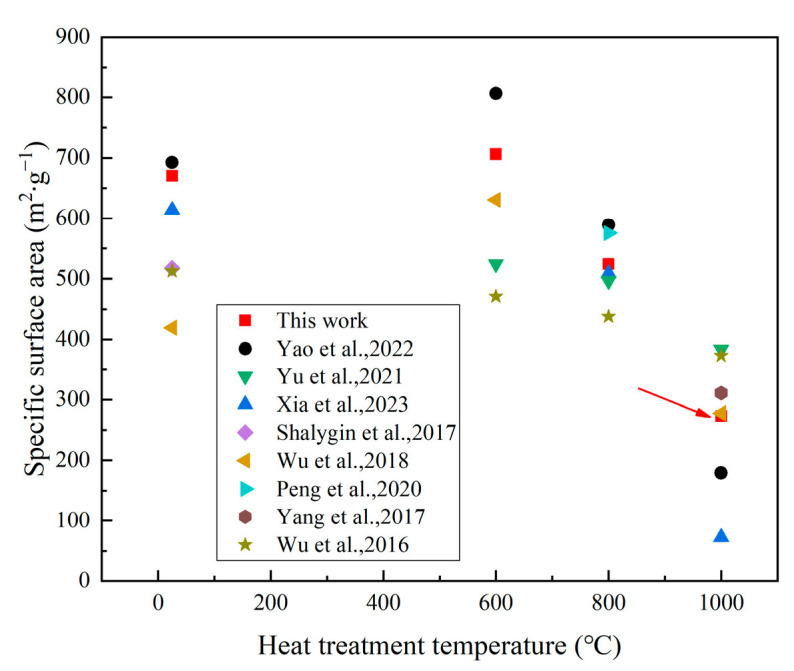
Comparison of specific surface area at different temperatures with that reported in the literature. The corresponding references for each piece of data are: Yao et al., 2022 [11]; Yu et al., 2021 [32]; Xia et al., 2023 [33]; Shalygin et al., 2017 [34]; Wu et al., 2018 [35]; Peng et al., 2020 [36]; Yang et al., 2017 [37]; Wu et al., 2016 [38].

We compared the specific surface area of our study’s samples with those reported in the literature [11,32,33,34,35,36,37,38], as shown in Figure 9. The specific surface area of the samples prepared in our work is relatively high. Notably, despite substituting a portion of TEOS with acidic silica sol, the specific surface area after high-temperature calcination remains higher than that of some samples prepared using expensive organosilicon sources and supercritical drying processes.

**Figure 10:**
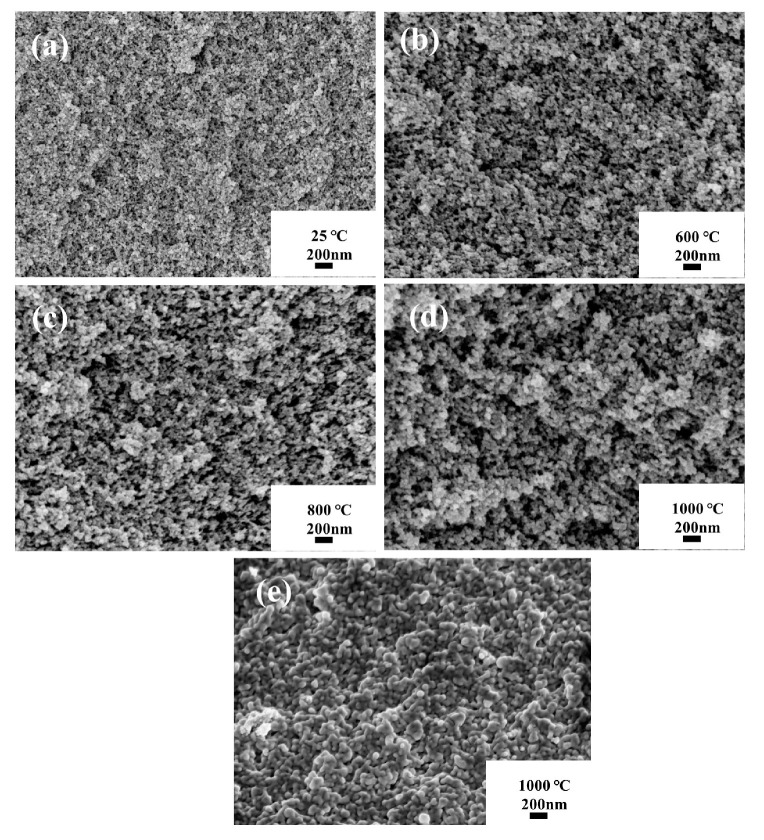
SEM images (**a**–**d**) of ASA-0.02 treated at different temperatures; SEM image (**e**) of SA-0.8 treated at 1000 °C.

Figure 10a–e present SEM images of individual samples at different temperatures. At room temperature, ASA-0.02 exhibits a complex 3D network skeleton structure with a uniform nanopore size distribution. Following calcination at 600 and 800 °C, a slight enlargement of pore size is observed, but the sample maintains the same structure as at 25 °C. Even at 1000 °C, the pore size of ASA-0.02 becomes larger. In addition, at this temperature, the network skeleton of the sample begins to loosen, and larger pores become evident. Some neighboring particles stack together, forming larger clusters. Nevertheless, the ASA-0.02 sample retains its typical complex 3D network nano-porous structure. In stark contrast, Figure 10e highlights the apparent sintering of the SA-0.8 sample at 1000 °C. Small particles disappear, giving rise to larger clusters, and the 3D network structure experiences severe disruption. This stark contrast indicates that the high-temperature resistance of SiO_2_ aerogels can be significantly enhanced with the addition of aluminum sol.

**Figure 11:**
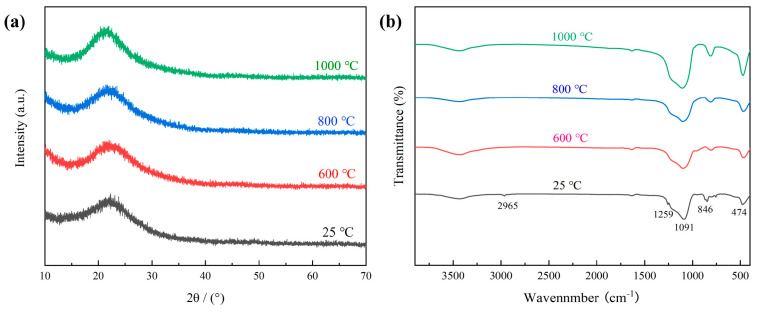
XRD patterns(**a**) and FTIR spectra(**b**) of ASA-0.02 calcined at different temperatures.

The XRD patterns of the untreated blank sample and the ASA-0.02 aerogel after calcination at different temperatures are presented in Figure 11a. Notably, the high temperature did not induce any significant alteration in the crystal structure of the samples. ASA-0.02 exhibited a consistent amorphous structure even after exposure to elevated temperatures. Concurrently, the intensity of the diffuse peaks representing SiO_2_ between 20–30° increased with the rise in calcination temperature. This may be attributed to the oxidation of hydrophobic groups on the surface of the skeleton to active Si-OH at elevated temperatures, while an increased formation of Si-O-Si occurs through dehydration condensation reactions between Si-OH at high temperatures. Importantly, no mullite crystals were observed at 1000 °C, a phenomenon reported in the literature. The preceding analysis establishes that a portion of the aluminum in the aluminum sol incorporates into the SiO_2_ lattice, enhancing the thermal stability of the aerogel and impeding crystalline transition. Another fraction of the aluminum element exists in the form of boehmite, which undergoes decomposition at high temperatures. However, due to its minimal content, the XRD patterns did not reveal the presence of the Al_2_O_3_ crystal phase [39].

We conducted additional FTIR analysis on the ASA-0.02 sample, and the findings are illustrated in Figure 11b. The corresponding -CH_3_ peaks at 2965 cm^−1^, 1259 cm^−1^, and 846 cm^−1^ vanish upon calcination at temperatures exceeding 600 °C. This disappearance is attributed to the oxidation of -CH_3_ to Si-OH at elevated temperatures, leading to an augmentation in the intensity of the Si-OH absorption peak at 960 cm^−1^ at 600 °C. However, at 800 °C, the intensity of the Si-OH absorption peak diminishes. This phenomenon is a result of the dehydration and condensation reaction between Si-OH, resulting in the formation of Si-O-Si bonds at high temperatures. The concomitant rise in the intensity of the Si-O-Si absorption peaks corresponding to 1091 cm^−1^ and 474 cm^−1^ at 1000 ℃ can be attributed to this process. Furthermore, owing to the low aluminum content in the ASA-0.02 sample, distinctive peaks of boehmite were not observed in the FTIR pattern.

**Figure 12:**
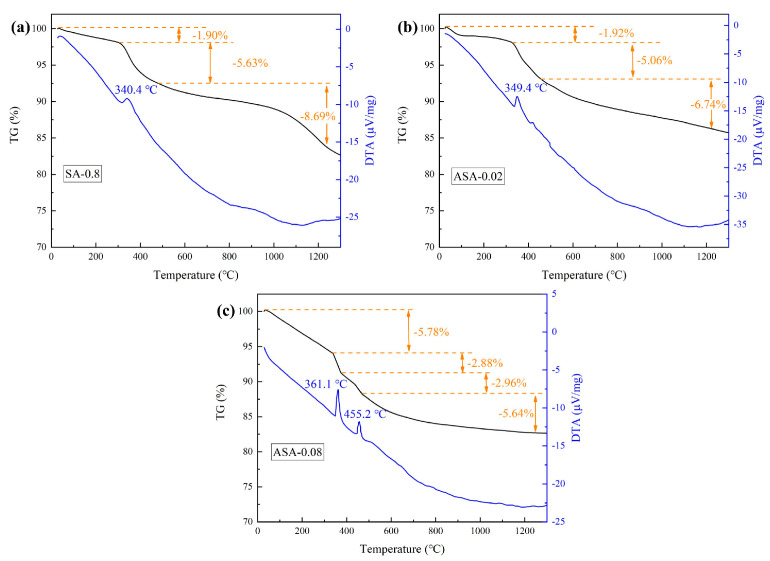
TG–DTA curves of (**a**) SA-0.8, (**b**) ASA-0.02, and (**c**) ASA-0.08.

Figure 12a–c displays TG and DTA curves for SA-0.8, ASA-0.02, and ASA-0.08. The TG curves of the SA-0.8 sample reveal three distinct stages. The initial stage, spanning from room temperature to 300 °C, results in a total mass loss of 1.90%, attributed to the evaporation of residual organic matter and water in the sample. In the second stage, occurring between 300 °C and 420 °C, a mass loss of 5.63% is observed, accompanied by an exothermic peak at 340.4 °C in the DTA curve. This peak is attributed to the oxidation of Si-CH_3_ groups on the hydrophobically modified surface of the aerogel skeleton, transforming into Si-OH. The third stage, which occurs from 420 to 1300 °C, involves the oxidized Si-OH undergoing a condensation reaction, leading to the formation of the Si-O-Si bond and resulting in a total mass loss of 8.69%. Similar to SA-0.8, the TG curves for ASA-0.02 are divided into three stages. In comparison to SA-0.8, the mass loss of the ASA-0.02 sample is similar in the first two stages but decreases to 6.74% in the third stage, accompanied by a flattening of the TG curve above 1000 °C. This suggests that the ASA-0.02 sample exhibits higher stability at high temperatures than SA-0.8. The ASA-0.08 sample shows a new heat absorption peak at 455.2 °C with a higher total mass loss. FTIR analysis in Section 3.4 reveals the presence of aluminum elements in the aerogel skeleton in the form of boehmite (γ-AlOOH) after the addition of alumina sol. The content of γ-AlOOH increases with the alumina sol content, leading to a higher mass loss in ASA-0.08 compared to the ASA-0.02 sample, as γ-AlOOH undergoes dehydration to γ-Al_2_O_3_ with increasing temperature.

**Figure 13:**
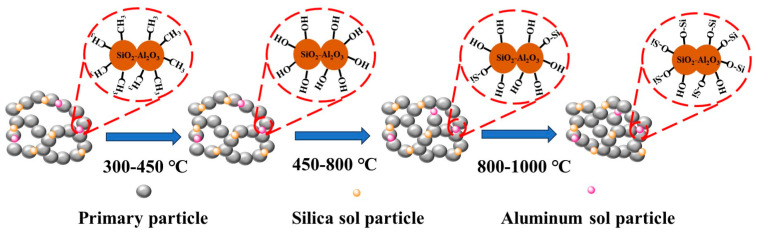
Schematic diagram of the skeleton structure during the sintering process of the ASAs.

Combined with the above discussion, we can summarize that the change in the ASA material at high temperatures can be divided into three steps, as shown in Figure 13. The first step is from 300 to 450 °C. The temperature increase will make the -CH_3_ on the surface of hydrophobically modified aerogel oxidized to Si-OH. At this time, the aerogel has hydrophobicity to hydrophilicity, and this process does not have much effect on the porous structure of the sample skeleton. In the second step from 450 to 800 °C, a dehydration condensation reaction between Si-OH occurs at high temperature, and SiO_2_ particles diffuse into the neck region. This necking causes some of the pores in the original aerogel skeleton to decrease, and the increase in neighboring SiO_2_ particles causes the particles to grow to form larger clusters. In the third step from 800 to 1000 °C, the SiO_2_ particles continue to increase in size and the clusters in the skeleton increase significantly, at which point some of the pore structure is destroyed. However, since we used a composite silica source, the addition of acidic silica sol and aluminum sol provided a large number of nucleation sites for the nucleation process, which improved the strength and stability of the aerogel skeleton. Thus, the driving force for diffusion of aerogel particles at high temperatures was reduced. On the other hand, after the addition of aluminum sol, some aluminum elements entered into the SiO_2_ skeleton to form Si-O-Al bonds, which led to electrostatic repulsion on the surface of the aerogel particles and effectively suppressed the necking effect between the particles [11]. In addition, the presence of Si-O-Al bonds inhibited the occurrence of phase transition, which was also the reason for the good thermal stability of the ASA samples.

## 4. Conclusions

In conclusion, the successful preparation of an aluminum-doped silica aerogel (ASA) with superior thermal stability and favorable formability was achieved using the sol-gel method and atmospheric pressure drying, utilizing TEOS as well as acidic silica sol as a composite silicon source and aluminum sol as an inorganic aluminum source. A comprehensive discussion on the effects of the molar ratio of acidic silica sol to TEOS, Al/Si molar ratio, and calcination temperature on ASAs provided detailed insights. Specifically, when maintaining the molar ratio of acidic silica sol to TEOS at 0.8, the resulting sample exhibited a specific surface area of 683.204 m^2^·g^−1^. Concurrently, the introduction of aluminum sol played a crucial role in enhancing the high-temperature resistance of the sample. The ASA with an Al/Si molar ratio of 0.02 consistently maintained higher specific surface areas at 600 °C (705.956 m^2^·g^−1^), 800 °C (524.267 m^2^·g^−1^), and 1000 °C (273.099 m^2^·g^−1^) compared to SA without the addition of aluminum sol. The analysis of the ASA’s heat-resistant mechanism revealed that the presence of acidic silica sol and aluminum sol particles contributed significantly by providing numerous nucleation sites during the gel process. Additionally, a portion of aluminum from the aluminum sol entered the SiO_2_ framework, effectively inhibiting phase transitions at high temperatures. In summary, the results demonstrate that aerogels with excellent heat resistance can be efficiently prepared utilizing low-cost acidic silica sol as a substitute for certain organosilicon sources and employing atmospheric pressure drying. This approach holds considerable promise for advancing the application of aerogels in large-scale production.

## Figures and Tables

**Table 1:** Gelation time, density, and pore structure characteristics of SA with different molar ratios of acidic silica sol to TEOS.

Sample	Gelation Time (min)	Bulk Density (g·cm^−3^)	Specific Surface Area (m^2^·g^−1^)	Pore Volume (cm^3^·g^−1^)	Average Pore Diameter (nm)
SA-0	73	0.117	579.540	3.110	21.456
SA-0.2	61	0.125	706.659	2.612	14.787
SA-0.4	56	0.132	743.898	2.766	14.873
SA-0.6	51	0.135	754.243	2.806	14.880
SA-0.8	55	0.138	683.204	3.090	18.094
SA-1.0	65	0.159	585.139	3.140	21.467
SA-1.2	81	0.178	540.383	2.648	19.598

**Table 2:** Gelation time, density, and pore structure characteristics of ASAs with different molar ratios of Al/Si.

Sample	Gelation Time (min)	Bulk Density (g·cm^−3^)	Specific Surface Area (m^2^·g^−1^)	Pore Volume (cm^3^·g^−1^)	Average Pore Diameter (nm)
SA-0.8	55	0.138	683.204	3.090	18.094
ASA-0.02	67	0.142	670.432	2.507	16.747
ASA-0.04	68	0.147	624.340	2.304	14.761
ASA-0.06	79	0.154	598.462	2.188	14.624
ASA-0.08	92	0.166	580.844	2.162	14.890
ASA-0.1	115	0.187	570.316	1.397	11.016
ASA-0.12	125	0.223	526.521	1.620	12.307

**Table 3:** The specific surface area of ASAs with different Al/Si molar ratios at high temperature.

Sample	Specific Surface Area (25 °C)/m^2^·g^−1^	Specific Surface Area (600 °C)/m^2^·g^−1^	Specific Surface Area (800 °C)/m^2^·g^−1^	Specific Surface Area (1000 °C)/m^2^·g^−1^
SA-0.8	683.204	545.145	240.195	16.082
ASA-0.02	670.432	705.956	524.267	273.099
ASA-0.04	624.340	686.276	519.568	277.328
ASA-0.06	598.462	670.238	517.435	236.236
ASA-0.08	580.844	675.452	519.191	220.475
ASA-0.1	570.316	557.016	518.883	203.088
ASA-0.12	526.521	489.963	480.081	209.856

## Data Availability

Data are available from a publicly accessible repository.
